# Kinetic pupillary size using Pentacam in myopia

**DOI:** 10.3389/fnins.2022.981436

**Published:** 2022-11-25

**Authors:** Kaixiu Li, Xiaoqi Li, Qun Wang, Liqiang Wang, Yifei Huang

**Affiliations:** ^1^Medical School of Chinese People’s Liberation Army (PLA), Beijing, China; ^2^Department of Ophthalmology, The First Medical Center, Chinese PLA General Hospital, Beijing, China; ^3^Department of Ophthalmology, The Third Medical Center, Chinese PLA General Hospital, Beijing, China

**Keywords:** high myopia, myopia, pupil light reflex, autonomic nervous system, arousal, Pentacam

## Abstract

**Purpose:**

To compare if the kinetic pupillary changes differs between high myopia (HM) and low/moderate myopia by Pentacam.

**Setting:**

Chinese People’s Liberation Army (PLA) General Hospital, Beijing, China.

**Design:**

Comparative study.

**Methods:**

In this cross-sectional retrospective study, 44 eyes of 44 patients were recruited in the Refractive Surgery Center of Chinese PLA General Hospital. Eyes were divided into two groups according to the refractive error: low/moderate myopia (22 eyes; −2.99 ± 1.09 D) and HM (22 eyes, −12.93 ± 3.44 D). At the beginning of the experiment, we made trials of scanning one false pupil by Pentacam. All patients underwent the Pentacam examination three times. Pupillary diameters (PD) during the scan process and other parameters were measured using the Pentacam. Coefficient variations of PD (CV) during the different scanning periods were analyzed comparatively between the two groups.

**Results:**

Pentacam once time output 25 Scheimpflug images, with 13 ones during the period from 1st to 1.5th s and 12 ones during the period from 2.5th to 3rd s after the scanning onset. For the spatial order on all the 25 meridians, 13 Scheimpflug images came out when the Pentacam rotated from 60° to 153°meridians and the remaining 12 Scheimpflug images from 161° to 245° meridians. Among pupillary parameters, no statistical significance existed in PD25, PD13, and PD12 (pupil diameter’s mean from all, former 13 and remaining 12 of 25 Scheimpflug images, respectively) (*P* > 0.05) between the two groups. However, there were statistically significant differences in CV25 and CV13 (coefficient variation of the pupil diameters from all and former 13 of 25 images, respectively) (*P* < 0.001), with no statistical significance in CV12 (coefficient variation of the pupil diameters from remaining 12 of 25 images) between both groups.

**Conclusion:**

Twenty-five Scheimpflug images on Pentacam had the temporal and the spatial orders. CV in eyes with HM was lower than that in eyes with low/moderate myopia in a certain period of the Pentacam scan. Kinetic pupillary size in HM changed more slowly than that in low/moderate myopia during some scanning period analogous to the phasic response of the pupil reflex.

## Introduction

High myopia (HM) estimates from 2000 to 2050 suggest significant increases in prevalence globally, with almost 1 billion people with HM by 2050 (9.8% of the world population) (Holden et al., 2016). A vast majority of myopia cases are characterized by excessive axial elongation of the eye over time. In some communities with a high prevalence of HM, myopic macular degeneration has been found to be the most frequent cause of irreversible blindness (Iwase et al., 2006; Xu et al., 2006).

A few studies alleged that an autonomic imbalance could be a precursor to axial elongation and result in myopia (Gilmartin et al., 2002; Chen et al., 2003). The size of the pupil at any one time reflects the relation between the opposing sympathetic (dilator) and parasympathetic (constrictor) influences (Smith, 1992; Prettyman et al., 1997). The sizes of the pupils change continuously in response to variations in ambient light levels to regulate the amount of light entering our eyes, and this process is known as the pupillary light reflex (PLR) (Lowenstein and Loewenfeld, 1950; Toates, 1972). The kinetic parameters of the PLR favor us to dissect the relative contributions of both influences.

The recently introduced anterior segment analysis system, Pentacam (OCULUS, Wetzlar, Germany), is based on a high-resolution rotating Scheimpflug camera (De Bernardo and Rosa, 2018), which takes a maximum of two seconds to generate a complete image of the anterior eye segment (Oculus). This instrument is widely used in the daily clinical practices of ophthalmology. It provides access to collecting and storing personal digitized ocular anterior segment records in a completely standardized and controlled manner. The data from the Pentacam system allows for the integrated assessment of pupillary size changes. In the dark environment, it uses a red LED light to find the corneal apex automatically and a blue slit light source with a wavelength of 475 nm to illuminate the eye. Thus, it can be speculated that the Pentacam system could imitate the process of PLR. One of the major strengths of Pentacam is that it is a high-resolution system with a minimum resolution of 5 μm on Scheimpflug images, which is more accurate than known pupillometers (Bergamin and Kardon, 2002; Naber et al., 2013; Vartanian et al., 2015; Yuan et al., 2021). Moreover, to prevent accommodation-induced pupil alteration, imaging was done during monocular fixation of an afocal, illuminated pinhole-like target by a red light-emitting diode (Demer, 2016).

Since myopia is multifactorial, we wanted to investigate the relationship of the kinetic pupillary changes with the refractive error using Pentacam. To the best of our knowledge, there is hardly any study analyzing the possible effect of HM on pupil kinetics. Therefore, this study aimed to (1) analyze the spatial and the temporal orders of Pentacam’s 25 Scheimpflug images of the pupil, and (2) tentatively explore the characteristics of the pupillary alteration with in eyes with HM using the Pentacam.

## Materials and methods

This retrospective, non-randomized consecutive case comparison study adhered to the principles of the Declaration of Helsinki. The institutional review board approved all experimental procedures of the Chinese People’s Liberation Army (PLA) General Hospital, Beijing, China. Written informed consent was routinely obtained from each participant before the refractive surgery to use their clinical data.

### Patient selection

Medical records of patients undergoing ICL/TICL implantation or corneal refractive surgery at Chinese PLA General Hospital were reviewed between 1 August 2020 and 1 April 2022.

Exclusion criteria were: (1) a best-corrected visual acuity (BCVA) of no more than 40/50 in either eye, (2) poor quality of Pentacam scan, (3) angle kappa on the X- or the Y-position >0.20 mm, (4) asymmetrical pupils, meaning the difference between the horizontal diameter and the vertical diameter centered on corneal apex or pupil center of the iris image >0.1 mm, (5) systemic conditions with known ocular involvement, systemic medication with known central nervous system effects and neurologic and psychiatric illness. Finally, 44 eyes of 44 patients were available for analysis. Twenty-two individuals with low/moderate myopia were defined as ones with spherical equivalent (SE) between −5.0 and −0.5 D (Group A). As controls, 22 patients with bilateral HM with SE ≤−6.0 diopters (D) or an axial length >26.5 mm (Group B) were evaluated in the same way.

All patients underwent complete ophthalmic examinations; namely, visual acuity testings, slit-lamp examination, intraocular pressure (IOP) measured using a tonometer (Canon Full Auto Tonometer TX-F; Canon, Tokyo, Japan), corneal topography (Pentacam, Oculus Inc., Wetzlar, Germany), endothelial cell density (ECD) (SP. 2000P; Topcon, Tokyo, Japan), biometry measurements (IOLMaster700, Carl Zeiss AG, Oberkochen, Germany), fundoscopy, and ultrasound biomicroscopy (UBM) (BME-300, MEDA, Tianjin, China) for ICL/TICL implantation.

### Pentacam examination

In a dark, sound-attenuated room, all subjects underwent Scheimpflug imaging three trials for each eye by a trained technician. Pupil sizes recorded in the first two trials were removed from analysis to control for confounding effects on pupil size due to transient onset responses and because observers needed some time to become oriented after trial onset (Naber et al., 2013). Therefore, the eye of the less angle kappa or more round pupil with quality specifications showing “OK” on the third test was used to analyze. Participants were dark-adapted for 5 min before testing. After a random duration of 6–8 s central fixation, participants were required to maintain steady fixation for a 4.0 s of Pentacam 3D Scan period. The scanning interval was about 8–10 s.

As for one trial in detail, it took 4.0 s for the 3D Scan mode to rotate two circles (4 × 180°) and generate 25 Scheimpflug images of the anterior eye segment. To determine the spatial and temporal orders of these 25 images, we made seven trials of scanning one false pupil with a stop time interval of 0.5 s. We placed it at approximately the same position as the participants’ pupils during the Pentacam recording. The datasets of images from the false pupil were used to transform 25 Scheimpflug images recorded from real participants into actual pupil spatial and temporal sequences.

**FIGURE 1:**
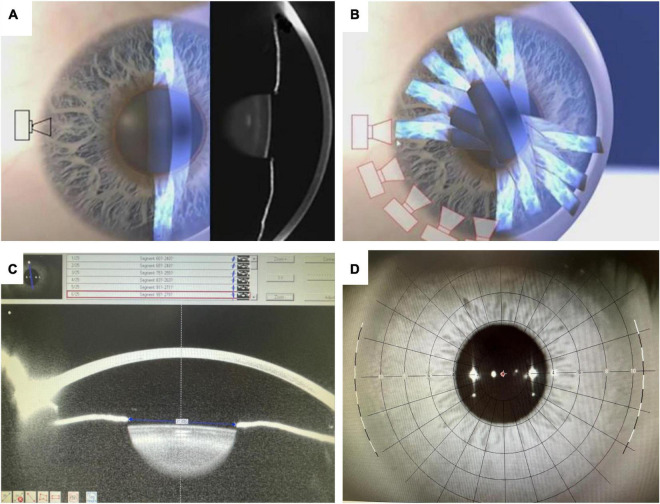
The data acquisition of Pentacam HR system **(A,B)**, PD from one of 25 Scheimpflug images on the 99° meridian **(C)**, and the iris image **(D)**: The cross indicating the center of the pupil and the red circle the apex of the cornea.

We used the right eye orientation for consistency. When the left eye was selected, it was flipped about the vertical axis to its mirror image right eye orientation. The pupil diameter (PD) returned by 25 Scheimpflug images represented the distance between two points on the diagonal line of the pupil on 25 meridians using the Pentacam customized software (Pentacam Version 6.08r27, OCULUS^1^) (Figure 1; Oculus). In addition, white-to-white (WTW), kappa-X and kappa-Y (the X- and the Y-position of the pupil center relative to the cornea’s apex, respectively), PDAH and PDAV (pupil diameter centered on the corneal apex in the horizontal and vertical direction of the iris image, respectively), PDCH and PDCV (pupil diameter centered on the pupil center in the horizontal and vertical directions of the iris image, respectively) were also measured and recorded. Like other forms of ocular anterior segment imaging and Scheimpflug photography, one concern was a distortion of the structures by their optical qualities. Overcoming the distortion needed the application of de-warping software, which was performed automatically on the Pentacam.

### Statistical analysis

All statistical analyses were performed using SPSS 26.0 software (IBM, Armonk, NY, USA). The normality of all data was first checked using the Shapiro–Wilk-test. Coefficient variation (CV) was calculated for all the 25, the former 13, and the latter 12 pupillary variables in both groups. Unpaired *t*-tests or Wilcoxon signed-rank-tests were used to compare data between both groups. *P* < 0.05 was considered statistically significant.

## Results

**FIGURE 2:**
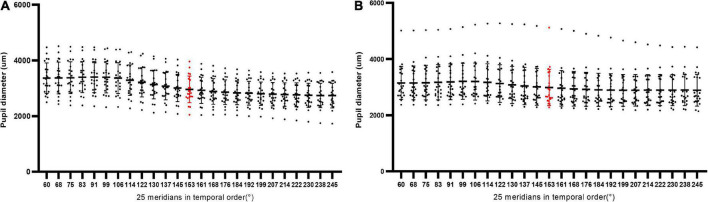
The scatter plots of PD25, PD13, and PD12 in all eyes of Group A (*n* = 22) and Group B (*n* = 22). The red part referred to the component from the 13th Scheimpflug image on the 153° meridian.

Forty-four eyes of 44 patients with low/moderate myopia (Group A, 22 eyes) and HM (Group B, 22 eyes) were included. The characteristics of the included eyes are displayed in Table 1 and Figure 2. The mean ages of the patients included in Group A and Group B were 31.23 ± 6.13 years and 31.09 ± 7.87 years, respectively. The mean axial length of Group A and Group B was 24.51 ± 0.59 and 28.72 ± 2.26 mm, respectively. The mean SE of Group A and Group B was −2.99 ± 1.09 and −12.93 ± 3.44 D, respectively. The mean PDAH of Group A and Group B was 3.94 ± 0.55 and 3.78 ± 0.67 mm, respectively. The mean PD25 of Group A and Group B was 3.04 ± 0.48 and 3.02 ± 0.60 mm, respectively (Table 1).

**TABLE 1:** Characteristics of ocular measurements in Group A and Group B.

	Group A (*N* = 22)		Group B (*N* = 22)		*P*-value
			
	Mean ± SD	*n*/22−*n*	Mean ± SD	*n*/22−*n*	
Sex (male/female)	–	14/8	–	11/11	0.543
Age (years)	31.23 ± 6.13	–	31.09 ± 7.87	–	0.949
Eye laterality (left/right)	–	7/15	–	9/13	0.531
PDAH (mm)	3.94 ± 0.55	–	3.78 ± 0.67	–	0.397
PDAV (mm)	3.98 ± 0.57	–	3.80 ± 0.67	–	0.332
PDCH (mm)	3.94 ± 0.55	–	3.79 ± 0.67	–	0.408
PDCV (mm)	3.99 ± 0.57	–	3.80 ± 0.66	–	0.333
SE (D)	–2.99 ± 1.09	–	–12.93 ± 3.44	–	<0.001*
AL (mm)	24.51 ± 0.59	–	28.72 ± 2.26	–	<0.001*
WTW (mm)	11.75 ± 0.40	–	11.68 ± 0.40	–	0.603
Kappa-X (mm)	–0.02 ± 0.09	–	0.02 ± 0.09	–	0.155
Kappa-Y (mm)	0.04 ± 0.07	–	0.07 ± 0.08	–	0.232
PD25 (mm)	3.04 ± 0.48	–	3.02 ± 0.60	–	0.891
CV25 (%)	8.98 ± 1.89	–	4.77 ± 1.44	–	<0.001*
PD12 (mm)	2.81 ± 0.43	–	2.90 ± 0.57	–	0.529
CV12 (%)	3.08 ± 1.53	–	2.38 ± 1.37	–	0.116
PD13 (mm)	3.28 ± 0.54	–	3.14 ± 0.64	–	0.195
CV13 (%)	4.78 ± 1.51	–	2.73 ± 1.37	–	<0.001*

*N*, number; D, diopters; PDAH and PDAV, pupil diameter centered on the corneal apex in the horizontal and vertical direction of the iris image, respectively; PDCH and PDCV, pupil diameter centered on the pupil center in the horizontal and vertical directions of the iris image, respectively; SE, spherical equivalence; AL, axial length; WTW, white-to-white; Kappa-X and Kappa-Y, the X- and the Y-position of the pupil center relative to the cornea’s apex, respectively; PD25, PD13, and PD12, pupil diameter’s mean from all, former 13 and remaining 12 of 25 Scheimpflug images of the anterior eye segment, respectively; CV25, CV13, and CV12, coefficient variation of the pupil diameters from all, former 13 and remaining 12 of 25 images, respectively. *P < 0.05 were considered statistically significant.

**FIGURE 3:**
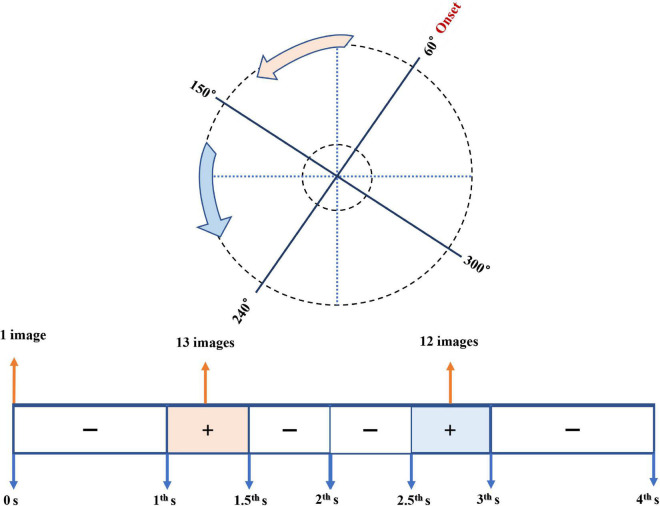
Diagram of the spatial and temporal orders of collected images using Pentacam. The upper plot showed the spatial order on the meridians centered on the apex of the cornea; the lower one referred to the temporal order along the timeline of the scan procedure. The pink and the blue parts meant the spatial and temporal sequences of the 13 Scheimpflug images and the remaining 12 ones, respectively.

Through trials of scanning one false pupil, the spatial and temporal orders of collected images were clarified (Figure 3). As for the temporal orders along the timeline of the scan procedure, only three periods of the whole scan process output images: (1) the very onset of the scanning (0 s) output the iris image, (2) the period from 1st to 1.5th s after the onset: 13 Scheimpflug images, and (3) the period from 2.5th to 3rd s: the remaining 12 Scheimpflug images. As to the spatial order on all the 25 meridians centered on the apex of the cornea, 13 Scheimpflug images undergone from 60° to 153° meridians, and the remaining 12 Scheimpflug images from 161° to 245° meridians.

**FIGURE 4:**
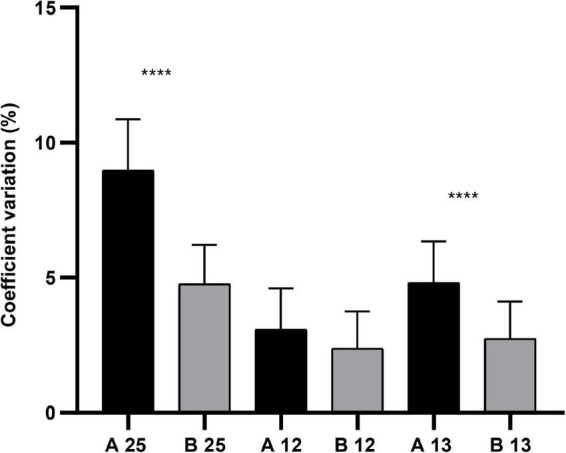
The comparison of CV25, CV13, and CV12 in Group A (*n* = 22) and Group B (*n* = 22), **** meant *P* < 0.001.

Among ocular measurements from Scheimpflug images, no statistical significance lay in PD25, PD13, and PD12 (*P* > 0.05; Table 1) between Group A and Group B. There were statistically significant differences in CV25 and CV13 (*P* < 0.001; Table 1), with no statistical significance in CV13 between both groups (Figure 4). According to the parameters of pupil sizes on the image of the iris, no significant differences were found for PDAH, PDAV, PDCH, and PDCV between the two groups. WTW, Kappa-X, and Kappa-Y showed no significant difference between the two groups.

## Discussion

The present study was the first consecutive case comparison study designed to investigate the kinetic pupillary changes of eyes with HM. In our research, the fixation red light of the Pentacam system flickered at 3.0 Hz, which could induce measurable pupillary oscillations (Miller and Thompson, 1978; Naber et al., 2013; Yuan et al., 2021). Additionally, there was a timespan of 6–8 s for redlight fixation preparation before the scanning onset, which meant the switch on of the blue light illumination. Hence, PD measured from the iris image at 0 s could not be judged as the baseline value of the PLR. The latency of the direct reflex of the normal eye ranged from 100 to 284 msec (Kase et al., 1984; Stamper et al., 2002), which was followed by the initial contraction of the pupil with no more than 2 s to peak constriction (phasic response) (Kardon, 1998; Adhikari et al., 2015). Therefore, we alleged that the period of 13 Scheimpflug images belonged to the phasic response period. However, as for the period of the remaining 12 Scheimpflug images, possibly from one part of the phasic response, pupil escape, or sustained period, it was complicated to figure out to which component it belonged. Thus, the 13 pupillary parameters from 13 Scheimpflug images were of analytical value. We substituted CV25 of the PD25 from all 25 Scheimpflug images for the velocity of pupil constriction calculated from only 2 Scheimpflug images with maximum and minimum pupil diameters. The reasons were as follows: one referring to a fixed time interval (40 ms) between two adjoining images instead of video recordings of pupillary changes, another concerning the pupil’s continuous oscillations known as hippus, taken into consideration during measurement. Hippus was present except at the extreme values of pupil constriction or dilation, where the magnitude of responsiveness was attenuated by mechanical constraints (Winn et al., 1994). In this study, the index of CV13 helped to control for the variability to parallel the pupil velocity during the period of 0.5 s.

This study demonstrated that several parameters of the PLR in HM were significantly different from those in low/moderate myopia. In cases of eyes with HM, the waveform shape of the pupil movement dropped slowly and gently. To the best of our knowledge, only one clinical study commented on the miosis speed of PLR in eyes with HM, which concluded that miosis speed was found to be correlated to the refractive error between −7.5 and 0 D, being slower for myopia (Ghoushchi et al., 2021). And the reasons for the discrepancy in miosis speed were not discussed in that study.

Why, then, would the eyes with HM have the lower CV25 and CV13? In this regard, the phasic response period of the PLR expressed an abruptly accelerated contraction of the pupils caused by a series of synaptic activities among the ganglion cells, their recipient neurons in the pretectal olivary nucleus in the midbrain, and an efferent output to the iris. Primarily, the iris, as the effector organ, receives a dual sympathetic/parasympathetic innervation (Theofilopoulos et al., 1995). One drug and nerve stimulation experiment on humans and animals demonstrated that sympathetic innervation was inhibitory, relatively small, slow, and augmented by concurrent levels of background parasympathetic activity (Gilmartin et al., 2002). Several pieces of the literature confirmed an autonomic imbalance that 30–40% of myopia individuals (not including HM ones) were likely to have access to a sympathetic inhibitory facility, which was lower than that of emmetropic ones (Strang et al., 1994; Gilmartin and Winfield, 1995; Gilmartin et al., 2002; Chen et al., 2003).

Interestingly, the parasympathetic activity had a very short onset latency to constrict the pupil [∼<270 ms with less than ∼800 ms to reach its extreme (Clarke et al., 2003; Wang and Munoz, 2014)]. By contrast, the pupil dilation caused by sympathetic activation arose slowly with an onset latency of ∼330 ms or more (often with a peak latency of more than 1 s) (Chapman et al., 1999; Steiner and Barry, 2011; Liao et al., 2016). Future work may elucidate further whether the difference between the sympathetic tone (the dark pupil response) and the parasympathetic activity (PLR) lies among low, moderate, and HM. Then, the PLR is not purely reflexive. Instead, the PLR can be modulated by the state of arousal (Loewenfeld, 1999), attention, high-level image perception, working memory, and other cognitive factor (Joshi and Gold, 2020). From these aspects of the related neural substrate perspectives, the possible relationship between myopia and PLR has not been investigated yet. HM was associated with structural and functional changes in the visual cortical area and non-visual cortices (i.e., altered neural substrate), one of whose clinical manifestations was the attention deficits (Zhai et al., 2016). Not surprisingly, the attention deficits were associated with the state of arousal (Brown and McMullen, 2001; Huang et al., 2019). It is prospective and hopeful that future studies involved in interdisciplinary cooperation are planned to confirm, enrich and explain our findings.

There are limitations in the current study. First, it utilized a relatively small sample size for a few numbers of patients satisfying strict enrollment criteria. Second, only two groups of the refractive error were analyzed because of enrollment criteria, especially for HM patients. We found that as the degree of myopia increased, the value of Kappa-X or Kappa-Y on Pentacam was often more than 0.2 mm when screening clinical cases for this study. Third, the methods and results of our research were controlled strictly and came out to record the partial process of the phasic response period of PLR. Future studies should be conducted with larger sample size and experimental methodology refinements to present more robust evidence of a link between myopia and PLR.

## Conclusion

In conclusion, the study is the first literature reporting the spatial and temporal orders of Pentacam’s 25 Scheimpflug images. It is possible to record the changes of PD at consecutive scanning time points using the Pentacam with high resolution. CV25 and CV13 in eyes with HM were lower than those in eyes with low/moderate myopia.

## Data availability statement

The raw data supporting the conclusions of this article will be made available by the authors, without undue reservation.

## Ethics statement

The studies involving human participants were reviewed and approved by the Ethics Committee of Chinese PLA General Hospital Review Board (Beijing, China). The patients/participants provided their written informed consent to participate in this study.

## Author contributions

KL and XL performed the initial clinical database search and completed the statistical analysis. KL produced the first draft of the manuscript and figures. YH supervised the study and contributed to the final approval of the version sent for approval. All authors contributed to the study revision and edited the manuscript.

## Footnotes

1
http://oculus.de
